# Six-membered-ring inorganic materials: definition and prospects

**DOI:** 10.1093/nsr/nwaa248

**Published:** 2020-09-28

**Authors:** Gang Liu, Xing-Qiu Chen, Bilu Liu, Wencai Ren, Hui-Ming Cheng

**Affiliations:** Shenyang National Laboratory for Materials Science, Institute of Metal Research, Chinese Academy of Sciences, Shenyang 110016, China; School of Materials Science and Engineering, University of Science and Technology of China, Shenyang 110016, China; Shenyang National Laboratory for Materials Science, Institute of Metal Research, Chinese Academy of Sciences, Shenyang 110016, China; School of Materials Science and Engineering, University of Science and Technology of China, Shenyang 110016, China; Shenzhen Geim Graphene Center, Tsinghua-Berkeley Shenzhen Institute and Shenzhen International Graduate School, Tsinghua University, Shenzhen 518055, China; Shenyang National Laboratory for Materials Science, Institute of Metal Research, Chinese Academy of Sciences, Shenyang 110016, China; School of Materials Science and Engineering, University of Science and Technology of China, Shenyang 110016, China; Shenyang National Laboratory for Materials Science, Institute of Metal Research, Chinese Academy of Sciences, Shenyang 110016, China; School of Materials Science and Engineering, University of Science and Technology of China, Shenyang 110016, China; Shenzhen Geim Graphene Center, Tsinghua-Berkeley Shenzhen Institute and Shenzhen International Graduate School, Tsinghua University, Shenzhen 518055, China

**Keywords:** six-membered-ring (SMR) materials, SMR concept, SMR physics, SMR chemistry, SMR mechanics, SMR classifications

## Abstract

The six-membered ring (SMR) is a common structure unit for numerous material systems. These materials include, but are not limited to, the typical two-dimensional materials such as graphene, *h*-BN, and transition metal dichalcogenides, as well as three-dimensional materials such as beryllium, magnesium, MgB_2_ and Bi_2_Se_3_. Although many of these materials have already become ‘stars’ in materials science and condensed-matter physics, little attention has been paid to the roles of the SMR unit across a wide range of compositions and structures. In this article, we systematically analyze these materials with respect to their very basic SMR structural unit, which has been found to play a deterministic role in the occurrence of many intriguing properties and phenomena, such as Dirac electronic and phononic spectra, superconductivity and topology. As a result, we have defined this group of materials as SMR inorganic materials, opening up a new perspective on materials research and development. With their unique properties, SMR materials deserve wide attention and in-depth investigation from materials design, new physical discoveries to target-wizard applications. It is expected that SMR materials will find niche applications in next-generation information technology, renewable energy, space, etc.

## Teaser

The authors defined SMR materials, which possess extensive six-membered-ring structural units and rich structural lattices, chemistry and physics, suitable for niche applications in next-generation information technology, renewable energy, space, etc.

## INTRODUCTION

**Figure 1.**
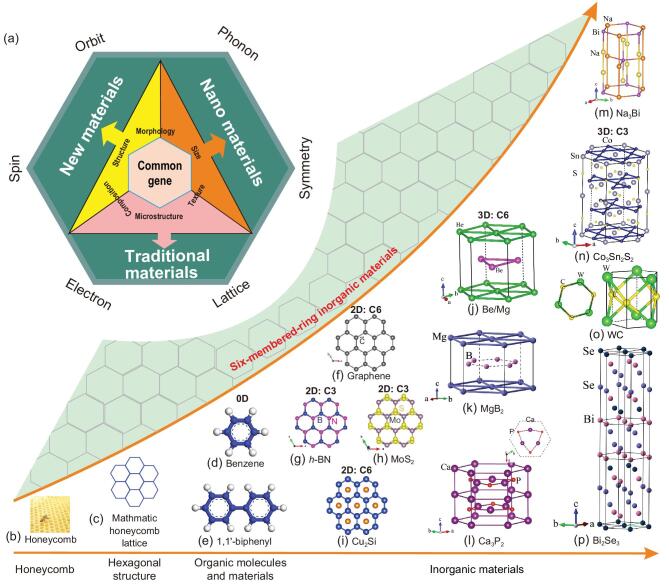
(a) Scheme of materials science and engineering research framework linked to different features. (b–p) The SMR structural unit from a bee's honeycomb (b) in nature to 0D organic molecules (d and e), 2D monolayer or layered materials (f–i) to 3D crystalline materials (j–p) with the SMR structural layers bonded by vdW force, ionic, metallic or covalent bonds along the *c*-axis. (f–i) Four typical 2D monolayer SMR materials of graphene, *h*-BN, the 2H phase of MoS_2_ and Cu_2_Si. (j–m) Four 3D SMR materials of Be/Mg, MgB_2_, Ca_3_P_2_ and Na_3_Bi. The C6-fold SMR structural layers are composed of pure metallic Be, Mg and pure boron atoms for MgB_2_, respectively. In both Ca_3_P_2_ and Na_3_Bi, their individual SMR layers comprise Ca/P and Na/Bi atoms, respectively, under the C3-fold rotation symmetry. (n–p) The 3D SMR materials of WC, Co_3_Sn_2_S_2_ and Bi_2_Se_3_ in which their C3-fold rotational SMR structural layers comprise W/C, Co/Sn and Bi/Se atoms, respectively. Their specified symmetry, space group and point group are summarized in Table 1.

Materials are basic to human society and are used to denote periods in history, such as the stone age and the iron age. To meet the requirements for rapidly increasing development, through exploration and utilization of materials, significant changes have occurred in composition, structure, size, morphology and texture (Fig. 1a). Identifying the true factors that govern the key properties of materials is essential for development of new materials. A honeycomb is a typical six-membered-ring (SMR) structure that exists extensively in nature (Fig. 1b), and such structures have found widespread uses in various fields, including materials science and engineering, architecture, transportation, mechanical engineering, chemical engineering, nanofabrication and biomedicine. In particular, a SMR honeycomb structure consisting of six carbon atoms is prevalent in organic chemicals (Fig. 1d) such as benzene, cyclohexane and pyrimidine, and exists in almost all drugs, such as artemisinin (Qinghaosu, 2015 Nobel prize), a novel therapy against malaria, and remdesivir, a potential treatment for the Corona Virus Disease 2019 (COVID-19) that is spreading all over the world.

The SMR honeycomb structure also exists extensively in many inorganic materials with diverse properties, ranging from metals, semimetals, semiconductors and insulators to superconductors and topological insulators. Graphene (Fig. 1f), a representative of two-dimensional (2D) materials, is a typical SMR material. It is constructed of a one-atom-thick SMR hexagonal lattice with hybridized *sp*^2^ covalent bonds of carbon atoms, which gives it various unusual properties, in particular, massless Dirac fermions and ultrahigh carrier mobility [1–3]. Changing the way two neighboring monolayers stack has led to exotic physics and intriguing properties [4–7]. For instance, AB-stacked bilayer graphene has a tunable bandgap, topological valley transport, tunable excitons and even-denominator fractional quantum Hall states. It is intriguing that by twisting two monolayer graphenes, the so-called ‘magic-angle bilayer graphene’ triggers a phase transition from a typical Dirac semimetal to a correlated Mott insulator and even to an unconventional superconductor at different twisting angles [8,9]. Many other layered 2D materials contain similar SMR structures. Another example of a 2D SMR material, transition-metal dichalcogenides (TMDCs, such as MoS_2_, WTe_2_ and MoTe_2_, see Fig. 1h) in their 1T structures exhibit large magnetoresistance,

pressure-driven superconductivity, a quantum spin Hall effect, Weyl semimetallic states, etc., because of strong spin-orbit coupling effects, and valley and exciton effects [10–15].

Among three-dimensional (3D) materials, the SMR periodic lattice exists not only in simple substances but also in wide range of compounds that show many unique properties. For example, metals such as Pt, Pd, Au and Ag, well-known catalysts with superior catalytic properties, have a periodic SMR structure in their energetically favorable (111) planes. Recently, pure beryllium metal (Be, Fig. 1j), which has a periodic SMR structure in its basal plane, has been found to possess a topological Dirac nodal line [16], which induces an anomalously large electron-phonon coupling strength on a Be (0001) surface [17] resulting from the presence of the topologically protected drumhead-like nontrivial surface states. The metallic diborides of MgB_2_ (Fig. 1k) with a periodic SMR in the pure boron layer form a typical BCS superconductor with a transition temperature of about 39 K [18], and, most recently, it was theoretically predicted to have unusual topological properties [19,20]. The van der Waals bonded Bi_2_Se_3_-type materials (Fig. 1p), an extensively studied family of both thermoelectric and topological insulators, also crystalize in SMR structural layers consisting of either Bi or Se atoms [21].

Besides those novel electronic properties, materials with SMR structural layers in both 2D and 3D systems usually have good mechanical properties, such as high strength, in combination with superior chemical stability, corrosion-resistance and high-temperature oxidation-resistance, especially in some oxides, borides and nitrides. Again taking graphene as an example, it is the strongest material ever examined, with a Young's modulus of ∼1.0 TPa and a strength of ∼130 GPa [4–7]. Furthermore, the SMR structure gives graphene a record high thermal conductivity, 10 times greater than copper, and the highest intrinsic electron mobility, about 100 times that of silicon. These excellent properties give SMR materials a very broad range of important applications in electronics, optoelectronics, spintronics, superconducting devices, quantum devices, topological devices for catalysis, energy storage and conversion, and many others.

Within this context, materials with a basic SMR structural unit are certainly a huge family, and many remain unexplored. Although many of those mentioned above have already become ‘stars’ in their own fields, little attention has been paid to the fundamental roles of the SMR structural unit across a wide range of different types of materials as it relates to their unique physical and chemical properties. In this article, we will try to identify the common features of SMR materials, build a bridge between the SMR ‘structural unit’ and properties of corresponding materials, and point out ways to find new members of the SMR material family with unusual properties and applications.

## THE CONCEPT OF SMR MATERIALS

To distinguish the above inorganic materials containing an SMR structural unit from others, we propose the concept of six-membered-ring inorganic materials (herein referred to as SMR materials). They are defined as a class of materials that have a SMR structural layer with three-fold (C3) or six-fold (C6) rotational symmetry as a basic unit. The bulk form of such materials is constructed of SMR structural layers bonded together by van der Waals (vdW), ionic, metallic or covalent bonds or with other atomic layers in 2D or 3D lattices. These materials have novel physical, chemical and mechanical properties that may lead to a revolution in the fields of information technology, energy technology, space applications, etc.

### SMR materials

On the basis of the above definition, for both 2D and 3D materials the occurrence of SMRs is usually associated with the C3 or C6 rotational symmetry (see Table 1). Figure 1f–i shows four typical 2D monolayer SMR materials, graphene, hexagonal boron nitride (*h*-BN), the most common 2H phase of MoS_2_ and monolayer Cu_2_Si. Both graphene and Cu_2_Si belong to the *P*6mm space group with a C6-fold SMR structure, whereas both *h*-BN and MoS_2_ crystallize in the P3m1 space group with a C3-fold SMR structure. Graphene, *h*-BN and Cu_2_Si have a flat monolayer structure, while a monolayer of MoS_2_ contains three layers of atoms (S-Mo-S). Monolayer Cr_2_Ge_2_Te_6_ and CrI_3_ have, respectively, been shown to have C6-fold Te and I SMR layers surrounding a central Cr atom [22–24]. Most recently monolayer MnBi_2_Te_4_, composed of seven atomic layers, has been synthesized, which has a C3-fold SMR structure [25–29]. All these 2D layers stack to form vdW-bonded 3D materials with many different large families with various stacking sequences and different spatial dimensions.

**Table 1.** Symmetries of some selected 2D and 3D SMR materials.

Compounds	Space Group	Point Group	Symmetries in Point Group

Graphene	P6mm	C_6v_	E; 2C6; 2C3; C2; 3${\sigma _v}$; 3${\sigma _d}$
Monolayer Cu_2_Si	P6mm	C_6v_	E; 2C6; 2C3; C2; 3${\sigma _v}$; 3${\sigma _d}$
Monolayer *h*-BN	P3m1	C_3v_	E; 2C3; 3${\sigma _v}$
Monolayer MoS_2_	P3m1	C_3v_	E; 2C3; 3${\sigma _v}$
Be, Mg	P6_3_/mmc	D_6h_	E; 2C6; 2C3; C2; 3C′2; 3C′2; I; 2S3; 2S6; ${\sigma _h}$; 3${\sigma _v}$; 3${\sigma _d}$
Na_3_Bi	P6_3_/mmc	D_6h_	E; 2C6; 2C3; C2; 3C′2; 3C′2; I; 2S3; 2S6; ${\sigma _h}$; 3${\sigma _v}$; 3${\sigma _d}$
MgB_2_	P6/mmm	D_6h_	E; 2C6; 2C3; C2; 3C′2; 3C′2; I; 2S3; 2S6; ${\sigma _h}$; 3${\sigma _v}$; 3${\sigma _d}$
Ca_3_P_2_	P6_3_/mcm	D_6h_	E; 2C6; 2C3; C2; 3C′2; 3C′2; I; 2S3; 2S6; ${\sigma _h}$; 3${\sigma _v}$; 3${\sigma _d}$
WC	P$\bar{6}$m2	D_3h_	E; 2C3; 3C′2; ${\sigma _h}$; 2S3; 3${\sigma _d}$
Co_3_Sn_2_S_2_	R$\bar{3}$m	D_3d_	E; 2C3; 3C′2; I; 2S6; 3${\sigma _v}$
Bi_2_Se_3_	R$\bar{3}$m	D_3d_	E; 2C3; 3C′2; I; 2S6; 3${\sigma _v}$

Besides these vdW SMR 2D and 3D materials, many other 3D materials certainly contain SMRs. Figure 1j–m shows four typical materials in which the SMR layers satisfy the flat planar C6-fold rotational symmetry, similar to graphene and *h*-BN. For instance, a SMR layer is found in pure beryllium, magnesium and titanium, a SMR boron layer exists in a very large family of AlB_2_-type materials [19,20,30] (e.g. MgB_2_ and TaB_2_), a SMR Na-Bi layer in the families of Na_3_Bi, K_3_Bi, and Rb_3_Bi [31–33], and a SMR Fe-Sn layer in the family of FeSn [34], as well as a distorted SMR Ca-P layer in Ca_3_P_2_ [35,36]. Figure 1n–p depicts three other typical 3D materials (WC-type family, Bi_2_Se_3_-family and Co_3_Sn_2_S_2_-family) containing SMR layers with C3-fold rotational symmetry. Stacked along the *c*-axis, these SMRs usually form 3D structures by sandwiching with other metallic or non-metallic layers via vdW, ionic or covalent bonds.

In the C6-fold SMR materials, the six atoms forming an SMR layer must be the same, whereas two types of atoms can form a C3-fold SMR layer. For graphene (Fig. 1f), beryllium (Fig. 1j), magnesium (Fig. 1j), monolayer Cu_2_Si (Fig. 1i) and MgB_2_ (Fig. 1k), the six atoms on the corners of the SMRs are the same. In the C6-fold SMR Na_3_Bi-family of materials, there are individual C3-fold SMR structural layers consisting of both Na and Bi atoms. Two individual C3-fold SMR structural layers mirror each other along the *c*-axis to form the whole C6-fold symmetry. Similarly, C6-fold Ca_3_P_2_ also holds an individual C3-fold SMR structural layer comprising both Ca and P atoms. In addition, C3-fold SMRs may consist of one type of atom (e.g. Co_3_Sn_2_S_2_ and Bi_2_Se_3_ in Fig. 1n and p), or two types of atoms (e.g. *h*-BN (Fig. 1g), MoS_2_ (Fig. 1h) and the WC-type family (Fig. 1o)).

Additionally, regardless of whether these SMR materials are monolayer materials, vdW layered materials or other 3D materials, (*s*,*p*)-block elements (mostly (*s*,*p*) non-metallic elements) seem necessary to create SMRs, either alone or in combination with other metallic elements. A possible reason is that (*s*,*p*) elements are necessary to produce *sp*^2^ hybridization, which is perhaps responsible for the stabilities of SMRs.

### Properties of SMR materials

#### 2D SMR materials

**Figure 2.**
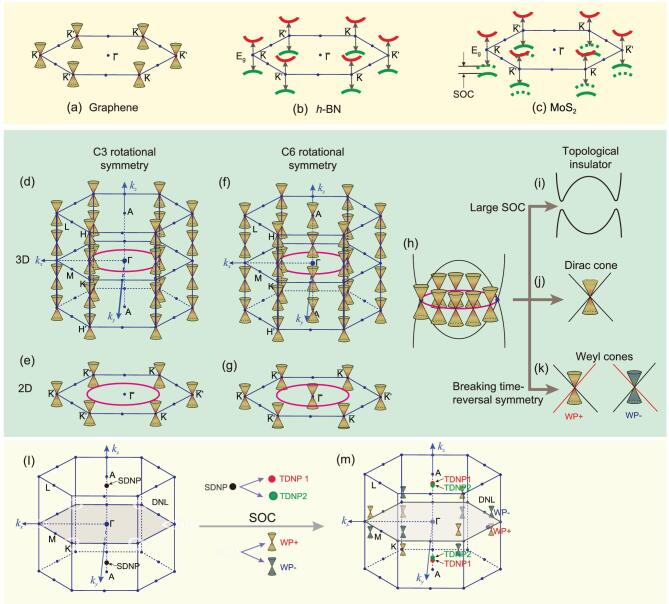
Schematic of possible electronic Dirac cone distributions in materials with SMR structural layers of C3 or C6 rotational symmetry. (a–c) Schematic electronic structures in 2D BZ for the monolayer materials of graphene, *h*-BN and the 2H phase of MoS_2_. (d and e) Dirac cones in 3D and 2D BZs of the C3 symmetry. (f and g) Dirac cones in 3D and 2D BZs of the C6 symmetry. (h) Dirac nodal line (or ring). (i) With strong spin-orbit coupling (SOC) interaction, a Dirac nodal line is split into a topological insulator. (j) With proper interactions among bonding, crystal field and SOC, a Dirac nodal line is degenerated into a Dirac cone, which is usually protected by symmetry. (k) With the time-reversal-symmetry breaking, a Dirac nodal line (or Dirac cone) is split into a pair of Weyl cones. In particular, if a Dirac nodal ring appears, it has to locate, if and only if, at the *k*_z_ = 0 plane surrounding the centered Γ point for both C3 and C6 symmetries. (l and m) 3D BZ for the non-centrosymmetric WC-family SMR materials. (l) Dirac nodal lines appear at the *k*_z_ = 0 plane surrounding the *K* point and the six degenerate nodal point (SNOP) along the Γ-A path for WC-family SMR materials without the SOC effect. By switching on the SOC effect, the Dirac nodal lines are broken into Weyl nodes and the SNOP is split into two triply degenerate nodal points (TDNPs) in panel (m).

The major representative of 2D SMR materials is graphene, which has typical *sp*^2^ electronic hybridization, where each carbon atom contributes its three *p* electrons to the degenerate *p*_x_, *p*_y_ orbitals to form planar covalent σ bonds with its three nearest neighbors. The π bonding electrons originating from the *p*_z_ orbital of each carbon form Dirac cones at the *K* or *K*′ points in the Brillouin zone (BZ) (Fig. 2a). This is mainly because in the unit cell of graphene, two carbon atoms obey non-equivalent translational symmetry but equivalent rotational symmetry, resulting in opposite phase factors of their wave functions at both the *K* and *K*′ points to form the four-fold degeneracy at the Fermi level. The three basic and important properties of graphene can be extracted from its SMR: (i) its electronic structure at low energies can be described by a massless Dirac-fermion model, leading to an extremely high mobility of carriers—as high as 15 000 cm^2^/(V·s) under ambient conditions and up to 250 000 cm^2^/(V·s) at a low temperature [7]; (ii) because of the covalent carbon-carbon σ bonding in its SMRs, graphene has the highest known mechanical tensile strength [37]; (iii) its Dirac phonons and Dirac nodal-line phonons [38] may be related with its large group velocities [39], and might contribute to the very high thermal conductivity [40].

Although monolayer *h*-BN [41–43] also crystallizes in an isoelectronic and isostructural SMR similar to that of graphene (Fig. 1f), its electronic structure is different. Within the SMR, the bond between neighboring B and N atoms is formed from the combination of B-*sp*^2^ and N-*sp*^2^ orbitals with a little ionic character from the different electronegativities of B and N. In particular, the number of the valence electrons is eight because of their combination of B-*s*^2^*p*^1^ + N-*s*^2^*p*^3^. Therefore, a semiconducting feature is expected between the conduction N-*p*_z_-like and valence B-*p*_z_-like orbitals, leading to a wide bandgap as large as 5.5–6.1 eV near the *K* or *K*′ points of its 2D BZ (Fig. 2b). This kind of electronic structure, together with the stable SMR, causes monolayer *h*-BN to be a wide-gap insulator with a high thermal conductivity, chemical inertness against high-temperature oxidation as well as acid-base stability for use in many functional devices. Furthermore, *h*-BN has been widely used as a superior 2D substrate to significantly improve the properties of other 2D materials it supports because of its atomically smooth surface that is relatively free of dangling bonds and charge traps.

Similar to *h*-BN, monolayer TMDCs of MoS_2_ with the most common 2H structure [44–58] (Fig. 1h) form a direct band-gap semiconductor with an *E*_g_ of ∼1.8 eV at the *K* or *K*′ points of its 2D BZ. This occurs mainly because of the presence

of the SMR structure (Fig. 2c) with the occurrence of the closed-shell configuration and the non-bonding *d* bands of Mo. Different from both graphene and *h*-BN, its Mo atom is a heavy metal element with a relatively large spin-orbital coupling (SOC) effect. It induces splitting of valence bands of the electronic valleys, corresponding to local energy minima, at two inequivalent moments of *K* and *K*′ in its BZ (Fig. 2c) to create spin-valley coupling interactions [47]. In particular, the spin splitting at the *K* and *K*′ valleys is coupled to be opposite with the presence of the SMR structure, leading to a unique feature where spin and valley degrees of freedom are coupled to each other. This is thought to open new ways to construct switching valleytronic devices, in parallel to spintronic devices, which manipulate the spin degrees of freedom of the electrons. TMDCs still have several polytopes, including the common 1*T* and 1*T*′ phases, and, importantly, they also contain ideal or slightly distorted SMR structural layers. It must be emphasized that the 2H phase of MoS_2_ is topologically trivial. However, it has been shown that monolayer MoS_2_ has a metastable 1*T*′ phase [48], in which the three planes of S-Mo-S atoms have rhombohedral stacking with a distorted SMR structure from spontaneous lattice distortion. Because of the strong SOC effect of the Mo atoms, this 1T′ phase has a fundamental bandgap (e.g. 0.08 eV around the Γ point of the 2D BZ for MoS_2_), together with the presence of the topologically non-trivial electronic structure. Thus, the 1T′ phase is a quantum spin Hall insulator, leading to helical edge states. With these structural modifications, a change of the coordination environment for the transition metal, and a change in its *d*-electron count, monolayer TMDCs have a large variety of properties, ranging from those of semimetals, semiconductors, and even to superconductors as well as topological insulators. For instance, one recent breakthrough called Ising superconductivity has been identified in TMDCs [49,50]. It has been revealed that Cooper pairs formed from carriers in *K* (*K*′) intrinsic spin valleys exhibit locked opposite spins and do not have responsibility to an in-plane pair-breaking field such as a magnetic field. This unique pairing-mechanism can enhance remarkably the in-plane upper critical field (*B*_c2_,//), which was first experimentally observed in ionic-gated MoS_2_ devices [49,50]. Similarly, in the superconducting monolayer NbSe_2_, an in-plane upper critical field *B*_c2_,//was reported to be more than six times larger than the Pauli paramagnetic limit [51], revealing unconventional Ising pairing protected by spin–momentum locking behaviors. Most recently, this unique behavior was observed in superconducting few-layer stanene, epitaxially strained α-Sn (111) [52] with the SMR structure, representing spin-orbit locking even at Γ point without participation of inversion symmetry-breaking. A Weyl semimetal [53,54] of the 2H monolayer SMR WTe_2_ exhibited anomalous giant magnetoresistance and superconductivity [55] and its distorted 1T′ structure was shown to be a large-gap quantum spin Hall insulator [56]. The quantum dots of SMR WSe_2_ are useful in quantum information processing [57] and photoelectrodes made of WSe_2_ are stable in both acidic and basic conditions, making them candidates for electrochemical solar cells [58].

The monolayer SMR material of Cu_2_Si [59–61] contains a flat and C6-fold Cu SMR with a center Si atom (Fig. 1i). Cu_2_Si is certainly metallic because of the non-closed-shell configuration. Interestingly, its stability depends on strong electronic hybridization between the center Si and the boundary Cu atoms of the SMR. Normally, the *p*_z_ orbital of Si would be unoccupied and the Cu-*d*-like orbital is in the full occupation of the *d*^10^ configuration. However, because of the C3-fold structure of the freestanding monolayer, the Cu *d*_3z-r^2^_ and d_x^2^-__y^2^_ states become partially unoccupied and the Si *p*_z_ orbital becomes occupied, thereby forming two different band inversions surrounding the Γ point as a result of the band crossing between the Si *p*_z_ and *d*_3z-r^2^_ (or d_x^2^-__y^2^_) states. The protection of the C6-fold and mirror symmetries thus results in two topological Dirac nodal lines surrounding the centered Γ point in the BZ (Fig. 2g). By following the most recent experimental observations of Dirac nodal lines [60,61], theory suggests that the monolayer Cu_2_Si has a superconducting temperature *T*_c_ of about 4.1 K [62].

**Figure 3.**
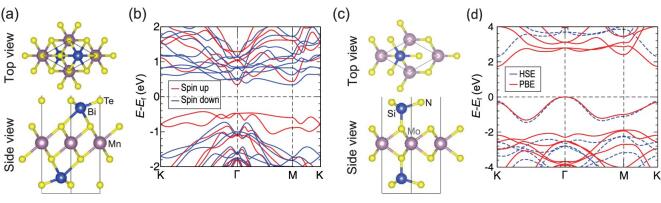
Lattice structures and electronic band structures of septuble-atomic-layer MnBi_2_Te_4_ (a and b) and MoSi_2_N_4_ (c and d) monolayer materials (adapted from Refs [27] and [71]). Blue curves and red curves correspond to conventional and hybrid first-principles calculations of density functional theory, respectively.

A vdW MnBi_2_Te_4_ SMR material has attracted extensive interest. It crystallizes in a rhombohedral layered structure with the space group R$\bar{3}$m [25], comprising septuble-layer blocks (Fig. 3a) stacked along the [0001] direction and inter-blocks bound to each other via vdW force. Both theoretical calculations and experimental results reveal that it is an antiferromagnetic topological insulator with a narrow direct bandgap of about 0.18 eV at Γ point (Fig. 3a) [25,63–69]. In particular, within the single layered septuble-layer block ferromagnetic ordering exists, antiparallel coupled between neighboring blocks in an out-of-plane easy axis. As expected, quantum anomalous Hall effect in this intrinsic magnetic topological insulator has been experimentally verified [25]. A high-Chern-number quantum Hall effect without Landau level also has been discovered in MnBi_2_Te_4_ [70]. Furthermore, by reducing its thickness from bulk to 2D form, various interesting topological states such as Weyl semimetal, Chern insulator and higher-order topological Möbius insulator have been realized [64–69].

Most recently, we discovered a new class of 2D vdW layered materials, the MoSi_2_N_4_ family [71], which have no experimentally known 3D parent crystals, and the monolayers of MoSi_2_N_4_ and WSi_2_N_4_ were synthesized using CVD. The monolayer of MoSi_2_N_4_ is built up by septuple atomic layers of N-Si-N-Mo-N-Si-N (see Fig. 3c), which also hosts a SMR structural layer comprising a MoS_2_-type MoN_2_ atomic layer. Although its structure is similar to that of MnBi_2_Te_4_ (Fig. 3), monolayer MoSi_2_N_4_ is a typical semiconductor with an experimentally measured bandgap of 1.94 eV, in accordance with the calculated values of 1.74 eV from conventional density functional theory and 2.25 eV from hybrid density functional theory. It also shows very high mechanical strength of ∼66 GPa and excellent ambient stability, which are much better than those of most semiconducting monolayers such as MoS_2_. Density functional theory calculations predict a large family of such monolayer structured 2D layered materials with all three components varying, which includes semiconductors, metals and magnetic half-metals, such as MoGe_2_N_4_, MoSi_2_As_4_ and VSi_2_N_4_ [71,72].

#### 3D SMR materials

*3D SMR materials with C6-fold symmetry.* There is a wide range of 3D SMR materials with C6-fold symmetry, from pure metals to compounds. Here, the focus is on only pure metals of beryllium and magnesium, superconducting MgB_2_ and topological Dirac semimetals of Na_3_Bi and Ca_3_P_2_.

(1) Beryllium and magnesium. Metallic Be and Mg crystallize in a hcp structure with a space group of P6_3_/mmc (Fig. 1j), in which Be or Mg can be viewed to form metallically bonded SMR layers stacked along the *c*-axis with C6-fold rotational symmetry. For a 3D SMR structure with C6 rotation symmetry, the lattice generally hosts mirror symmetries perpendicular and parallel to the C6 rotational axis. These symmetries ensure occurrence of the D_6h_ point group. Importantly, the crystalline structure of pure metals Be and Mg is a *P*6_3_/mmc space group, which means the electronic bands around the centered Γ point of the BZ have a D_6h_ point group. This point group splits the *p*_z_ and the degenerate

*p*_x,__y_ orbits because of the SMR structure, resulting in possible occurrence of electronic band inversion between the *p*_z_ and *s* orbits. Chemically, because Be has only *s* electrons, one would not expect electronic occupation of the *p* orbital. However, because of the crystal field effects, at the Γ point of its BZ an electronic band *s* → *p*_z_ inversion occurs, resulting in partial occupation of the *p*_z_ orbitals and some s-like orbitals becoming unoccupied. Once the band inversion occurs, these two bands cross each other and their crossings definitively form a closed loop around the Γ point without the spin-orbit coupling effect, and the two degenerate bands at any point on the closed loop have the opposite Berry curvatures under the *PT* symmetry. Therefore, their band crossings form a Dirac nodal line. The existence of *M*_z_ mirror symmetry of the D_6h_ point group mean that such a Dirac nodal line is forced to locate at the *K*_z_ = 0 plane surrounding the centered Γ point of the BZ. This kind of Dirac nodal line Dirac nodal rings not only surrounds the center Γ point of the *k*_z_ = 0 plane (Fig. 2f), but also exhibits a non-trivial topological electronic structure with *Z*_2_ index of (1; 0 0 0) [16]. Because the extremely weak SOC effect for both Be and Mg in combination with the *PT* symmetry is associated with the C6-fold rotation symmetry, the Dirac nodal lines are highly robust against various perturbations as long as their symmetries are maintained. This leads to non-trivial drumhead-like surface states on the (0001) surface [16], in agreement with previous photoemission spectroscopy observations on the Be (0001) surface. In particular, projection of the Dirac nodal line on the *k*_z_ = 0 plane in the BZ onto the (0001) surface shows a closed circle surrounding Γ, within which topologically protected non-trivial surface states appear. This discovery has resolved several long-standing (since the 1950s) anomalous issues regarding the Be (0001) surface, including severe deviations from the description of the nearly free-electron picture and giant Friedel

oscillations [16,73,74] and, most recently, the anomalously large electron-phonon coupling [17].

Because both Be and Mg have the SMR structural feature (Fig. 1j), it is easy to understand that Mg contains a topological Dirac nodal line in its *k*_z_ = 0 plane of the BZ and non-trivial drumhead-like surface states [16]. Some properties of Mg may be correlated with this unique electronic property. It is well known that, because of its light weight and high strength, Mg plays an important role in many industrial applications. However, pure Mg and its alloys are highly vulnerable to corrosion. Although this poor corrosion-resistance has been extensively investigated, its physical origin remains unclear. We speculate that the poor corrosion-resistance correlates with the topologically protected drumhead-like non-trivial surface states of Mg. From a chemical viewpoint, this type of state is highly localized, chemically active and also highly resistant to doping, adsorption, various defects and surface reconstruction [16].

In similarity to both Be and Mg, the transition metals Ti, Zr and Hf also crystallize in the C6-fold hcp SMR structure. We checked carefully their electronic band structures and saw similar Dirac nodal lines, which also exist in their bulk BZs when their SOCs are ignored. Furthermore, by switching on the SOC effects, their Dirac nodal lines slightly split because the atomic masses of Ti, Zr and Hf are much higher than those of both Be and Mg. However, it is a pity that around the Fermi level their Dirac nodal lines heavily overlap with the other *d*-like electronic orbitals and, therefore, the specific properties of the topology related to their Dirac nodal lines are, indeed, too complicated to be clearly distinguished from other trivial electronic states.

(2) MgB_2_. As emphasized above, metallic diboride AlB_2_-type materials have a large number of boron atom SMRs. For instance, the superconductor MgB_2_ [19] has an AlB_2_-type lattice with a space group of P6/mmm, in which Mg is located at the *Wickoff* site 1*a* (0, 0, 0) and boron occupies the 2*d* (1/3, 2/3, 1/2) site (Fig. 1k). Boron atoms form a C6-fold SMR structural layer with the point group C_3v_ at both *K* and *K*′ points of its 3D BZ, similar to graphene. Hence, there is no doubt that they share the same Dirac cones at both *K* and *K*′ points, given that every boron atom has *sp*^2^ electron hybridization with its three nearest neighbors. Because its valence electron number is less than that of carbon, the extra electrons from the metallic Mg atoms are required to fill the *p*_z_ orbital of boron to form π bonding electrons. In addition, because MgB_2_ is a 3D structure, any point on the boundary *H*′-*K*-*H* line of the 3D BZ has the same C_3v_ point group, leading to countless Dirac cones that form Dirac straight line states (Fig. 2d) traversing the whole BZ for both its electronic and phononic dispersions [19,20]. Importantly, Dirac cones exactly cross the Fermi level for graphene but those on the Dirac nodal straight line along the *H*-*K*-*H*′ line of MgB_2_ do not exactly cross the Fermi level. Some of them are above the Fermi level and form hole Dirac states, and some are below and form electron Dirac states. In addition, no topological electronic structure exists for the Dirac cone of graphene, while the Dirac nodal straight lines in the AlB_2_ family of materials are topologically non-trivial with the robust appearance of the topologically protected non-trivial surface states [19,20]. This also demonstrates the significant complementary role of a metallic element layer in modulating and enriching the properties of a pure non-metallic SMR layer.

There are many AlB_2_-type materials and almost all transition metals form such diborides with many novel properties, such as superconductivity, and electronic and magnetic properties. In addition, we must emphasize that they have other important properties related to the presence of the covalent SMR boron layer, such as excellent mechanical properties and thermal stability, and in particular, high hardness, high strength, good electrical and thermal conductivities, and excellent corrosion resistance, as well as extremely high melting temperatures (e.g. 3313 K for ZrB_2_ and 3523 K for HfB_2_). Therefore, they have been used as ultrahigh temperature ceramics in aviation and aerospace fields [75–77]. In contrast, the same binary Zr-B or Hf-B systems with lower or higher boron contents (ZrB, ZrB_3_, HfB_3_) do not have such outstanding high-temperature mechanical properties, because of a lack of the unique covalent boron SMRs.

(3) Na_3_Bi and Ca_3_P_2_. Ionically bonded SMRs also exist in a large number of 3D materials. Taking the typical compounds P6_3_/mmc-Na_3_Bi [32] and P6_3_/mcm-Ca_3_P_2_ [35,36] as examples (Fig. 1m and l), Na and Bi atoms form a flat ionically bonded SMR structural layer (similar to graphene), and Ca and P atoms form a distorted ionic SMR layer. They stack along the *c*-axis with the insertion of other Na (or Ca) atomic layers. Within the framework of the ionic picture, both Na_3_Bi and Ca_3_P_2_ could be expected to be semiconductors because of their closed-shell electronic configuration; however, they are metallic. For Na_3_Bi, the linear band crossings between Bi 6*p*-states and Na *s*-states along the *Γ*-*A* line in the BZ belong to two different irreducible representations distinguished by C3-fold rotational symmetry around the *Γ*-*A* axis associated with the SMR layers [32]. Without the SOC inclusion, a Dirac nodal line occurs. Nevertheless, the strong SOC effect of Bi splits its Dirac nodal line into two isolated 3D Dirac cones at (0, 0, $k_z^c \approx \pm 0.26\!\! \times\!\! \frac{\pi }{6})$ along the *Γ*-*A* path. As long as the symmetry of this SMR is retained, these Dirac cones will not be destroyed. Ca_3_P_2_ has similar physics for the presence of the Dirac nodal line, which lies in the *k*_z_ = 0 plane because of the band inversion between P *p*-states and Ca *d*_x^2^-__y^2^_ states around the centered Γ point. Of course, its occurrence is protected by both the rotational and mirror symmetries of the SMR structure [35]. Because SOC is negligible for the light elements Ca and P, its Dirac nodal line is stable at the Fermi level. Otherwise, Ca_3_P_2_ would become a semiconductor if there was a strong SOC. Importantly, both Na_3_Bi and Ca_3_P_2_ are topologically non-trivial. In the case of Na_3_Bi, the topology results in coexistence of both a 3D Dirac cone in its bulk phase and non-trivial topological surface states. This unusual feature, in contrast to conventional metals and topological insulators, has been confirmed experimentally [33,34], and the isolated Dirac cone behaves like a magnetic monopole in crystal momentum space, as experienced by a source or sink of Berry curvature, leading to an extraordinary magneto-transport property from the chiral anomaly [78,79]. Thus, its Fermi surface encloses two monopoles with opposite topological charge touching at the same *k*-point, indicating potential and intriguing applications of unusual quantum phenomena, physical and chemical properties. Later, the earlier characterized 3D topological Dirac semimetal P6_3_/mmc-Na_3_Bi [32] was found to be dynamically unstable, because the flat Na/Bi SMR layers induce large imaginary phonon frequencies. Distorting the SMR layers along the *c* axis results in stabilization of a new P$\bar{3}$c1 phase [80], as confirmed experimentally [81]. Because this buckled SMR layer still has C3-fold rotational symmetry, it shows exactly the same coexistence of the isolated 3D Dirac cones and a topological non-trivial electronic structure. In addition, the Na_3_Bi-type materials comprise a large family, mainly consisting of alkali metals and the main-group elements of oxygen, and exhibit many interesting properties from trivial semiconductors to Dirac semi-metals, because of the electronegativity difference and the strength of the SOC of their constituents.

*3D SMR materials with C3-fold symmetry.* There is also a wide range of 3D SMR materials with C3-fold symmetry. For example, Bi_2_Se_3_-type materials (Fig. 1p) are such a family including (a) those with excellent thermoelectric performance [82,83] and (b) topological insulators that have recently been studied extensively [84–86]. Here, we will focus on only WC-type and Co_3_Sn_2_S_2_-type materials.

(1) WC-type materials. Although the SMR materials mentioned above possess both *P* and *T* symmetries, some SMR materials with C3-fold symmetry have typical non-centrosymmetric structures (i.e. space group *P*6*mmn*) without the inversion center along the *z* axis. A typical example is the large family of WC-type 3D SMR *MX* materials (*M* = IVB, VB or VIB group transition metal elements, *X* = IVA, VA or VIA-group elements) [87–96], which crystallize in a non-centrosymmetric hexagonal structure, but with a planar C3-fold SMR layer consisting of three layers of atoms (*X*-*M*-*X*), as shown in Fig. 1o. We selected ZrSe as a prototypical example to show the crucial features of their electronic structures [93]. Without the SOC effect, two main features can be observed. First, a Dirac nodal line centered at each *K* point in the *K*_z_ = 0 plane of the BZ (Fig. 2l) is formed around the Fermi level because of the linear crossing of the inverted bands between the Zr *d*_xz_ + *d*_yz_ orbitals and the Zr *d*_x^2^-__y^2^_ + *d*_xy_ orbitals. Second, a six-fold degenerate nodal point (SDNP) occurs at (0, 0, *K*_z_ = 0.3025) along the Γ-A direction around the Fermi level because of another band inversion between the doubly degenerate Zr *d*_xz_ + *d*_yz_ and the Zr *d*_z^2^_-like orbitals at the A point of the BZ (Fig. 2l). Because the masses of both Zr and Se are not light enough for their SOC effects to be ignored, including them means that the electronic band structure exhibits changes around the Fermi level. First, because of the lack of inversion symmetry, spin splitting bands appear and each Dirac nodal line around the *K* point is broken into two Weyl points (WPs) with opposite chirality (e.g. WP+ and WP− as marked in Fig. 2m). In total, there are six pairs of WPs located at both *k*_z_ = ±0.01628 planes slightly above and below the *k*_z_ = 0 plane. All these 12 WPs have the same energy level. Second, the inclusion of SOC splits each SDNP into two triply degenerate nodal points (TDNP1 (0, 0, 0.2904) and TDNP2 (0, 0, 0.3146)) as marked in Fig. 2m along the Γ to A direction. Their appearance is protected by the *C*_3z_ rotation and mirror symmetries of the SMR structural layer. This kind of coexistence between the TDNPs and Weyl fermions was first predicted in WC-type SMR TaN material [87], but the TDNP was experimentally discovered first in the WC-type SMR, MoP [92]. Besides electronic TDNPs and Weyl fermions, for the first time it has been found that they have both phononic TDNPs and phononic WPs [93–95], and the phononic TDNPs of *MX* materials have recently been suggested to have potentially excellent contributions to their thermoelectric properties [96].

(2) Co_3_Sn_2_S_2_-type materials. The other materials that break the *PT* symmetry are SMR Co_3_Sn_2_S_2_ type [97–101]. Although they crystallize in a centrosymmetric structure, the presence of magnetic ordering breaks the time-reversal symmetry. As shown in Fig. 1n, Co_3_Sn_2_S_2_ has a rhombohedral structure of the space group R$\bar{3}$m (no. 166), in which a quasi-2D Co_3_Sn layer is sandwiched between sulfur atoms, and the cobalt atoms form a SMR layer surrounding the centered Sn atom in the hexagonal representation of this space group. In the quasi-2D Co_3_Sn layer, the cobalt atoms conform to the C_3z_ point group. In particular, Co atoms siting at the six corners of the SMR carry a ferromagnetic spin moment of 0.29 μ_B_/Co with a dominant out-of-plane magnetization with a Curie temperature of 177 K. DFT calculations show that the electronic bands corresponding to the spin-down channel are insulating, while the spin-up channel crosses the Fermi level and thus shows a metallic character. As expected, both magnetic ordering and the spin-orbital coupling effect split the linear crossing of the nodal lines in the spin-down channel of the electronic bands to finally form Weyl nodes [98]. Recently, Weyl fermions in Co_3_Sn_2_S_2_ have been experimentally confirmed to have Fermi arc states in a certain surface [99,100]. Therefore, Co_3_Sn_2_S_2_ is a Weyl ferromagnetic metal. The coexistence of Weyl fermions and ferromagnetic ordering in Co_3_Sn_2_S_2_ yields fascinating spin-electronic transport behavior, including a large intrinsic anomalous Hall effect with an anomalous Hall conductivity as large as 1130 Ω^−1^ cm^−1^, an order of magnitude larger than all known typical magnetic materials and systems [98]. In addition, the most recent experiments have observed a negative magnetoresistance that is consistent with the chiral anomaly expected from the presence of Weyl fermions close to the Fermi level [101].

## COMMON FEATURES OF SMR MATERIALS

The reason why we are defining SMR materials as a new materials class is to emphasize the importance of the SMR structure unit in determining their stabilities, physical and chemical properties, and possible uses. We shall now discuss the common features of materials that result from the SMR structure.

### Classification of SMR materials

**Figure 4.**
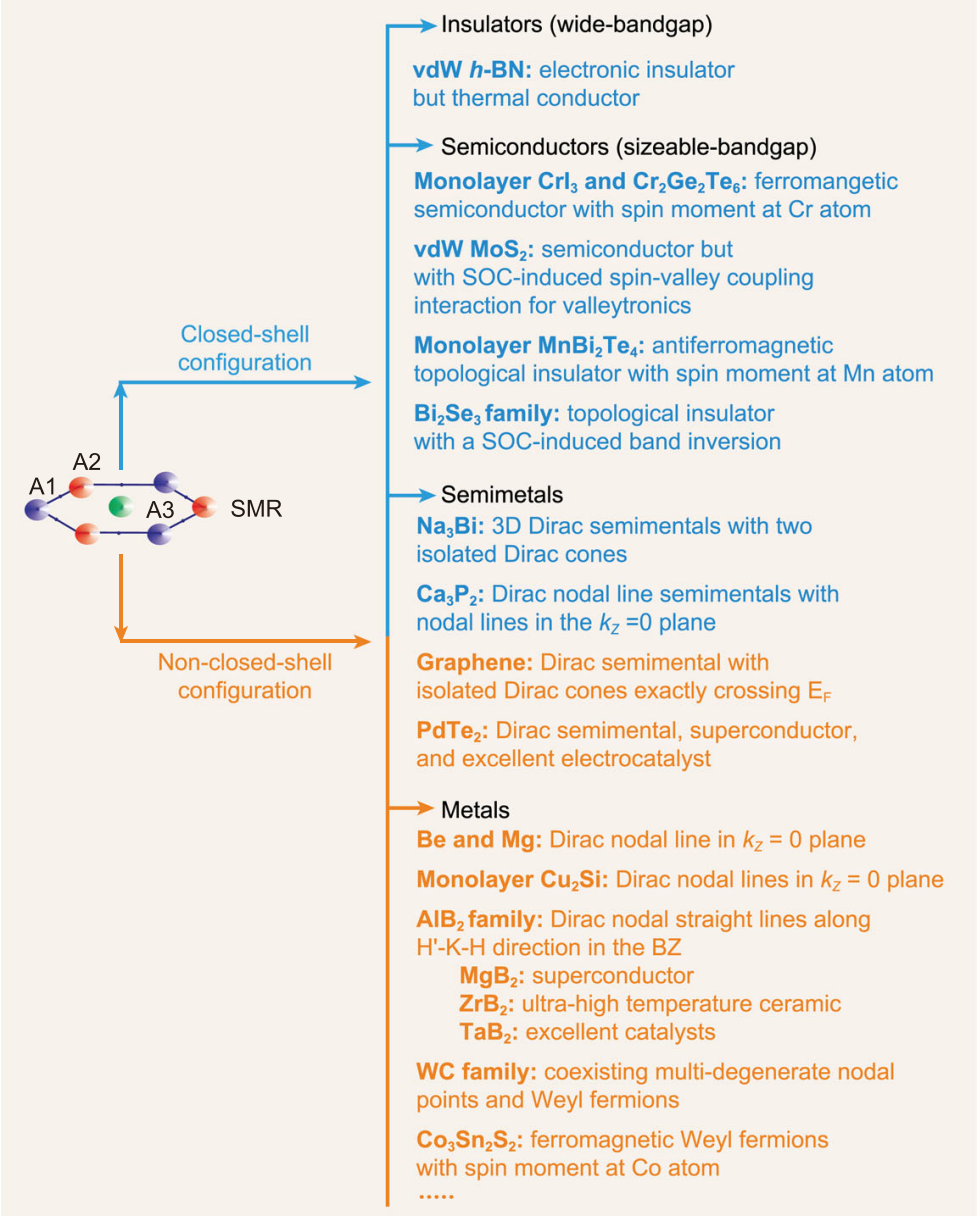
Linking from an SMR structural unit to SMR materials, and to excellent properties. Various SMR materials can be insulators, semiconductors, semimetals or metals with various exotic physical, chemical and mechanical properties, such as quantum phenomena, topological transport, spintronics and valleytronics, catalytic activities and corrosion/oxidation behaviors.

To classify SMR materials, we first need to understand the most basic SMR structure. As shown in Fig. 4, a SMR structure consists of A1 and A2 atoms with, in some cases, a central A3 atom. It is worth noting that these three atoms (A1, A2 and A3) can be either the same or different. As mentioned above, the SMR may consist of one, two or three elements. The A3 atom site can also be empty. The SMR can be either flat or buckled out of plane, and it usually comprises not only a single flat atomic layer, but also stacked atomic layers. There are, therefore, a large number of SMR structures.

Second, we shall consider SMR structures from the aspect of their electronic configurations. When the atoms in the SMRs have full electron shells, the materials can be wide-bandgap insulators, sizeable-bandgap semiconductors or semimetals. As illustrated in Fig. 4, monolayer *h*-BN is a wide-bandgap insulator, monolayer CrI_3_ and Cr_2_Ge_2_Te_6_ are ferromagnetic semiconductors with Cr atoms carrying the spin moment, Mn_2_Bi_2_Te_4_ is an antiferromagnetic topological insulator, the Bi_2_Se_3_ family have outstanding thermoelectric properties and are topological insulators, and monolayer MoS_2_ is a sizeable-bandgap semiconductor but with a SOC-induced spin-valley coupling interaction. The occurrence of bandgap opening, magnetic ordering and spin-valley coupling interactions correlates with the presence of SMR structures.

Of course, a closed-shell electronic configuration is also present in semimetal. For example, Na_3_Bi is a typical 3D topological Dirac semimetal, and Ca_3_P_2_ is a Dirac nodal line semimetal. However, the formation of semimetals does not need a closed-shell configuration, and, for example, graphene, without a closed-shell configuration, is a famous 2D Dirac semimetal. The main reason that graphene has Dirac cones at the Fermi level and the extremely weak SOC effect interacting with the SMR structure. If its SOC strength is theoretically increased to a certain value, its Dirac cone at both K and K′ momentum points splits to form a semiconductor. On the basis of this theoretical treatment, Kane and Mele made a pioneering theoretical discovery that predicted a quantum spin Hall effect by breaking the Dirac cone into a gap [102,103], introducing a new phase of quantum matter, later called topological insulators in HgTe [104,105], with the bulk material being insulating but the edges having a quantized, strong edge conductance.

When the constituents of the SMR have a non-closed-shell electron configuration, the materials basically are metallic with several unusual SMR-related features (Fig. 4). Among them, there is no doubt that Be and Mg are metals composed only of SMRs, and have a Dirac nodal line surrounding the centered Γ point with a topological non-trivial feature. This produces the drumhead-like non-trivial surface states which induce many anomalous properties, such as weak corrosion-resistance, high chemical activity, giant Friedel oscillations and an anomalously large electron-phonon coupling effect. In addition, AlB_2_-type materials are all metallic with various interesting properties, such as the superconductivity of MgB_2_, the ultra-high temperature thermal-resistance of ZrB_2_ and the superior catalytic performance of MoB_2_ and TaB_2_. Significantly, the SMR structure of the boron atomic layers plays a central role in understanding their unusual properties that correlate with the topological Dirac nodal lines and topological phonons.

Among the SMR metallic materials, Co_3_Sn_2_S_2_ is another large family of layered magnetic Weyl metals. One should point out that the magnetic ordering originates from the spin moment of Co atoms exactly locating at each corner of the SMR structure, which is the key to inducing Weyl fermions in its minority-spin electronic band structure. Interestingly, in monolayer CrI_3_ and Cr_2_Ge_2_Te_6_ ferromagnetic semiconductors, Cr atoms sitting at all six corners of the SMR have a spin moment.

### Unique SMR-determined momentum-contrasting physics

It should be emphasized that the C3/C6 rotational symmetry of SMR units is a crucial feature of SMR materials. The C3/C6-symmetry-related physics and chemistry may occur accordingly. One of the most unique properties is the occurrence of momentum-contrasting physics at the *K* (and *K*′) point of the hexagonal BZ corners. Any constituent in SMR materials has a certain SOC strength, either weak or strong, which induces the opposite spin splitting behaviors at *K* and *K*′ points, accompanied by momentum-contrasting Berry curvatures. This feature may induce occurrence of spin-valley contrasting physics [44–48] for SMR semiconductors with a certain SOC strength, and plays a crucial role in intrinsic Ising superconductivity [49–52] for SMR metallic materials. The former gives rise to the strong valley Hall effect flowing to opposite transverse edges when an in-plane electric field is applied and also leads to a strong valley-dependent optical selection rule at both *K* and *K*′ points. The latter induces large SOC splitting, which makes spins of Cooper pairs possibly aligned along the out-of-plane direction, accompanied by a large in-plane upper critical field exceeding the Pauli paramagnetic limit. Its superconductivity does not effectively respond to the in-plane magnetic field, which is a highly unique behavior different from conventional superconductors.

### SMR-related topological properties

In general, SMR materials with *s*,*p* non-metallic elements have degenerate *p*_x_,*p*_y_ electronic orbitals and the energy of the *p*_z_ orbital is determined by the interlayer interactions. The *p*_z_ energy is possibly higher or lower than the energy of the degenerate *p*_x_,*p*_y_ orbitals or the *s* orbitals. The kind of orbital-resolved splitting is a prerequisite for band inversion of SMR materials, which is key to producing a topological non-trivial electronic structure. If the energy of the *p*_z_ orbital is lower than its *s* orbital because of band inversion within the framework of the SMR structure, it is possible to form a Dirac nodal line or nodal ring, such as in Be, Mg, Ti, Zr, Hf and Ca_3_P_2_. If the electrons form closed-shell configurations as a result of formation of a planar *sp*^2^ or ionic framework, semiconductors are certainly expected (e.g. *h*-BN and MoS_2_). However, when the SOC strength for electronic band splitting is comparable to that of the bandgap [106], a topological non-trivial electronic structure possibly occurs [84–86,107]. For instance, with strong SOC, the orbitals are further split to form a topological insulator for Bi_2_Se_3_, a 3D topological Dirac semimetal for Na_3_Bi and a spin-valley coupling interaction for monolayer MoS_2_. Because a SMR structure generally has time-reversal and inversion symmetries, it is difficult to expect the occurrence of Weyl fermions. However, the ferromagnetic ordering can be stabilized by the Co atoms in the SMR ring in monolayer Co_3_Sn_2_S_2_, in which the spin moment of the Co atoms breaks the time-reversal symmetry to allow formation of topological Weyl fermions.

In addition, in terms of atomic vibrations producing phonon dispersions for materials with SMR structural layers, the phonon modes along both the *a* and *b* axes are degenerate. But it is possible for the phonon modes along the *c* axis to have a higher or lower frequency mainly because of interlayer interactions together with the atomic masses. With these interactions, their phonon spectra possibly contain a topological Dirac nodal-line and nodal-ring phonons. For instance, Dirac phonons not only exist at two inequivalent *K* and *K*′ points, but also appear on the Γ-L and Γ-K lines in the case of graphene and there still is a phononic nodal ring surrounding the central Γ point. These topological Dirac phonons and nodal-line phonons induce non-trivial edge states along both zigzag and armchair boundaries, and these are confined to the boundaries in one-way propagation, so are immune to backscattering.

In terms of the point groups and C3 or C6 symmetry of a SMR structure, we can draw three general conclusions from the above discussion (Fig. 2):

- Among C6-fold 3D SMR materials with mirror symmetry, Dirac straight line states occur not only along the *K* (*K*′)-*H* direction of the boundary but also possibly along the center *Γ*-*A* direction. If C6-fold 3D SMR materials are reduced to 2D ones in which the SMR structure still exists, single Dirac cones would perhaps occur at the *K* (*K*′) point or *Γ* point of the 2D BZ. Among C3-fold 3D SMR materials with mirror symmetry, Dirac straight line states are only allowed to appear along the *K* (*K*′)-*H* direction of the boundary of the BZ. Furthermore, if this kind of 3D SMR material with C3-fold symmetry is reduced to 2D, single Dirac cones would possibly only occur at the *K* (*K*′) point of the 2D BZ. As an example, the 3D AlB_2_-family SMR materials exhibit Dirac straight line states only along the *K* (*K*′)-*H* direction of the boundary of the 3D BZ in Fig. 2f, and the 2D graphene has Dirac cones at the *K* (*K*′) point of the 2D BZ.
- Once topological nodal rings exist in SMR materials, they must be located in the *k*_z_ = 0 or 1/2 plane of the BZ. Among 3D SMR materials, the Dirac nodal ring in Be, Mg and Ca_3_P_2_ appears only at the *k*_z_ = 0 plane surrounding the Γ point of the BZ in Fig. 2f, and, in the case of WC-type materials, the Dirac nodal ring occurs at the *k*_z_ = 0 plane surrounding the *K* (*K*′) point of the BZ in Fig. 2l. Among 2D SMR materials, the phononic Dirac nodal ring in graphene appears around the Γ point of the 2D BZ, and in a monolayer of Cu_2_Si, two electronic Dirac nodal rings occur around the Γ point of its 2D BZ.
- Once the C3 or C6 rotation symmetry is broken because of strain or their time-reversal symmetry is broken by a spin-polarizing effect or by a strong SOC effect, these Dirac cones, nodal lines or nodal rings will be split into topological insulator, Weyl fermion or Weyl nodal line states (e.g. the ferromagnetic Weyl semimetal of Co_3_Sn_2_S_2_ from magnetic ordering from Fig. 2h to Fig. 2k; the topological insulator of Bi_2_Se_3_ from a strong SOC from Fig. 2h to Fig. 2i; the 3D Dirac semimetal of Na_3_Bi from a strong SOC effect from Fig. 2h to Fig. 2j), etc.

Within this context, there are numerous topological properties of SMR materials. As shown in Fig. 4, in addition to wide-bandgap insulators or sizeable-bandgap (magnetic) semiconductors, almost all SMR materials are topologically non-trivial in their electronic or phononic bands. First, SMR structures are indeed a good platform to possibly host a topological nature for both electrons and phonons, including topological insulators and topological semimetals (e.g. Dirac and Dirac nodal line (or ring) semimetals, Weyl and Weyl nodal-line (or ring) semimetals, and high degenerate nodal point materials, etc.), as discussed in ‘Properties of SMR materials’ Section. Therefore, they almost all have physical properties possibly induced by topology, Berry curvature or a chiral anomaly. One of the most prominent electronic structures is the occurrence of topologically protected non-trivial surface (or edge) states (e.g. Fermi arc states in both Dirac or Weyl semimetals and drumhead-like states in topological nodal-line materials). Using topology, the quantum anomalous Hall effect was first discovered in magnetically doped topological insulator thin films of Cr_0.15_(Bi_0.1_Sb_0.9_)_1.85_Te_3_ [108] based on the parent SMR Bi_2_Se_3_ family. Second, the SMR structural unit provides a suitable model to capture the essence of the physics related to the unique six corners of the SMR. They not only have the topology to produce spin-locking helical states, but also induce interesting spin-valley coupling associated with the strong SOC strength and even other possible couplings, including spin-valley-lattice coupling and charge-spin-valley-lattice coupling.

### SMR-related chemical and mechanical properties

Besides the topological and physical properties of SMR materials discussed above, there are many other novel properties of SMR materials depending on their bonding schemes and electronic and phononic band structures, as summarized in Fig. 4.

Many *s*,*p* non-metallic elements form covalent SMR materials usually with ideal *sp*^2^ or quasi-*sp*^2^ hybridizations, for example, graphene and the AlB_2_-type family with ideal *sp*^2^ hybridization. The presence of a SMR structure bonded covalently to the nearest neighboring atoms produces several advantages, including high structural/thermal stability, high strength, high modulus, high fracture toughness, and high specific strength and specific stiffness, in particular, at high temperatures, in combination with good corrosion/oxidation resistance, as well as chemical inertness because the covalent bonding is electronically saturated. Although uses of some carbides and graphene at very high temperatures are limited because carbon is oxidized to CO and CO_2_ at over 700 K, some SMR materials, such as SMR borides, nitrides and oxides, can survive at extremely high temperatures because of their high melting points and the large cohesive energies of their strong covalent bonds. Some SMR materials retain their properties at ultra-high temperature conditions and are used as thermal insulating materials in hypersonic vehicles and aircrafts.

Some SMR materials (e.g. graphene and *h*-BN) consisting of *s*,*p* non-metallic elements have a high thermal conductivity because of their very high vibration frequencies, caused by their light atomic masses combined with the presence of Dirac phonons with an extremely high group velocity. In particular, covalent SMR materials with a closed-shell configuration are usually wide-bandgap insulators or sizeable-bandgap semiconductors, which together with their high thermal conductivity make them good in electronic insulating applications. In addition, some covalent SMR materials with good chemical inertness are not only used for good corrosion protection, but also have potential applications as building blocks to construct heterostructures and hybrid multilayers.

The topological nature of metallic or ionic SMR materials can give a combination of superior properties such as a stable supply of carriers, possible fast charge transfer and strong catalytic active sites, which are essential for various catalysis applications. Certainly, metallic or ionic SMR materials with active *s*,*p* metallic elements usually have weak corrosion and oxidation resistances.

Some vdW SMR materials are also common with only *s*,*p* non-metallic elements or in combination with metallic elements. These materials are usually ductile with weak interlayer interactions and weak resistance to slip, which makes them suitable as solid lubricants.

## TYPICAL PROMISING APPLICATIONS OF SMR MATERIALS

As shown in Fig. 4, SMR materials have many interesting properties related to the six-membered ring and many interesting potential applications. For instance, graphene has stimulated tremendous research activity because of its revolutionary impact on industry in many fields, such as energy storage, solar cells and nanoelectronics as well as superconductors, etc. For SMR topological materials, the range of potential applications is even more extensive, because of their thermodynamic properties, magnetic field dependences including Landau level quantification and chiral anomalies, and transport including the suppression of back scattering and linear response behaviors correlated with their large Berry curvatures of topological band structures, as well as topological superconducting behaviors including Majorana Fermi. Here, as examples, we discuss two kinds of representative applications to demonstrate utilization of the superior unique physical and chemical properties of SMR materials.

### 2D SMR materials for topological field effect transistors

**Figure 5.**
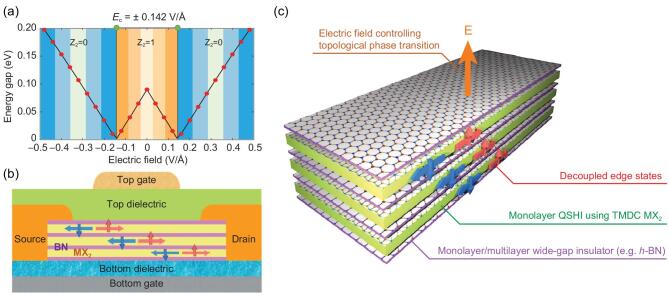
A topological field effect transistor (TFET) made of 2D van der Waals SMR heterostructures of 1T′-*MX*_2_ and dielectric layers. (a) Topological phase diagram of 1T′-MoS_2_ as a function of vertical electric fields. The critical field strength is ±0.142 V/Å, marked by two green dots. (b) Schematic of the TFET. The central component (c) is a heterostructure of alternating monolayer 1T′-*MX*_2_ and mono-/multilayer wide-bandgap insulators such as *h*-BN, with a horizontal width as narrow as ∼20 nm. Carriers (charge or spin) are injected from the source electrode and ejected into the drain electrode. The on/off switch is controlled by a vertical electric field through the top and bottom gates. Mono-/multilayer wide-bandgap insulators effectively screen the interaction between adjacent *MX*_2_ layers, preventing them from detrimental topological phase change and parametrically increasing the number of edge channels (reproduced with permission [48], Copyright 2014, The American Association for the Advancement of Science).

Graphene is a trivial Dirac semimetal with linear Dirac cones crossing the Fermi level. As early as in 2005, it was found that by enforcing an artificially large SOC effect within the Kane-Mele model, the Dirac cone of graphene splits into an extremely small gap, becoming a quantum spin Hall insulator [101]. We now understand that graphene with a large SOC effect is topologically non-trivial, with spin and charge transport in gapless edge states that propagate at the sample boundaries. If we return to the SMR materials discussed above, this phenomenon could very easily occur because many such materials have a large SOC effect. Among them, TMDCs like MoS_2_ become promising candidates for quantum spin Hall insulators, as discussed in ‘Properties of SMR materials’ Section. Their occurrence is protected from localization and elastic backscattering. This study also showed that the fundamental bandgaps of the monolayer 1T′ MoS_2_ phase can be tuned by an electric field, leading to a topologically trivial bandgap-closing transition for disappearance of the helical edge states. On the basis of the possibility of electrical control of the on/off charge/spin conductance of the helical edge states, Qian *et al.* proposed a topological field effect transistor (TFET, see Fig. 5) [108]. In the core of the TFET device in Fig. 5b and c, they used two different monolayer SMR materials: monolayer *MX*_2_ and a monolayer wide-bandgap SMR *h*-BN insulator that is electrically insulating to the adjacent *MX*_2_ layer. The *h*-BN layer protects parallel helical edge channels to avoid interlayer hybridization. This device can support dissipation-less charge/spin transport in the ‘on’ state (*Z*_2_ = 1), with a quantized conductance of 2*Ne*^2^/*h*, where *N* is the number of quantum spin Hall layers. The electric field indeed provides the driving force to switch on/off topological nature from this topological quantum spin Hall insulator (*Z*_2_ = 1), with helical edge states to a trivial insulator (*Z*_2_ = 0) and turning the edge conduction off (Fig. 5a) [108–110]. As a further support of these quantum spin Hall states, recent experiments confirmed clear indications of topological band inversion and

bandgap opening as well as edge conduction in the monolayer 1T′ phase of WTe_2_ [111–114].

### 3D SMR materials for water splitting catalysis

**Figure 6.**
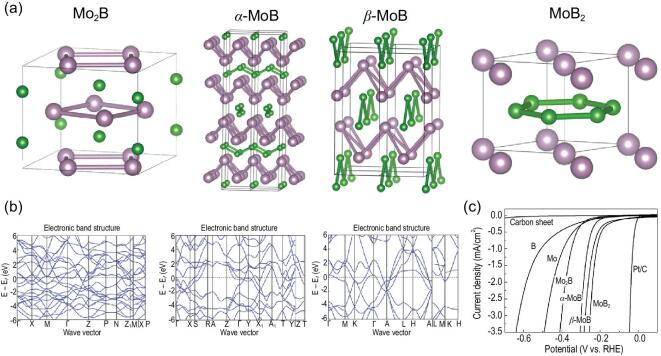
Linking from an SMR structural unit to the unique electronic structures and superior hydrogen evolution reaction of metal borides. (a) Crystal structures of four metal borides, Mo_2_B, α-MoB, β-MoB and MoB_2_, and (b) their electronic structures. (c) Polarization curves for amorphous B, Mo, Mo_2_B, α-MoB, β-MoB and MoB_2_ measured in 0.5 M H_2_SO_4_. IR-drop was corrected. Specific surface area: Mo_2_B: 9.09 m^2^/g; α-MoB: 3.97 m^2^/g; β-MoB: 15.27 m^2^/g; MoB_2_: 4.23 m^2^/g (reproduced with permission [118], Copyright 2017, John Wiley and Sons).

3D SMR AlB_2_-type materials have Dirac nodal lines, as illustrated above. Their Dirac nodal lines are usually located near the Fermi levels and induce strong non-trivial drumhead-like surface states. These topologically protected surface states in combination with the relatively high mobility associated with bulk Dirac nodal lines, make the materials suitable for chemical and catalysis applications [115–117], one of which is catalysis for water splitting. In an early experiment, the activity of the hydrogen evolution reaction (HER) of four molybdenum borides as electrocatalysts was compared (Fig. 6). It can be seen that hexagonal MoB_2_ with a boron SMR structural layer has a much superior HER activity to the other three borides (tetrahedral Mo_2_B, α-MoB and orthorhombic β-MoB) that do not have the boron SMR structure. This result is even more surprising given that MoB_2_ has the second smallest specific surface area of 4.23 m^2^/g [118]. We have anticipated this superior HER activity of MoB_2_ to the presence of the boron SMR structural layer which is closely correlated with the Dirac nodal lines. As illustrated in Fig. 6b [119–121], below the Fermi level two linear crossings appear, which indicate the occurrence of Dirac nodal lines because of the C3-fold rotational and mirror symmetries. The crossing points are slightly split into a tiny gap because of the relatively small SOC strength of Mo. Resistant to other localizations and perturbations, the drumhead-like non-trivial surface states induced by the Dirac nodal lines possibly highlight the exceptional ability for electron transfer to protons, as conceptually proposed for a topological quantum catalyst [117], which may provide an interesting insight for further clarification. In fact, this case is not the only indication of the advantage of SMR materials for catalysis. Much superior catalytic activity was observed in the case of TaB_2_ with the same boron SMR layer structure as hexagonal MoB_2_, when it was compared with Pt as an HER co-catalyst with Ta_2_O_5_ [122]. There is no doubt that TaB_2_ also has Dirac nodal lines and its induced drumhead-like non-trivial surface states accelerate electron transfer. Another ferromagnetic Weyl SMR material, Co_3_Sn_2_S_2_, was shown to have excellent electrocatalytic activity for water oxidation, because of the (0001) surface non-trivial Fermi arc states induced by the Weyl fermions near the Fermi level of its bulk phase [123]. As illustrated in ‘3D SMR materials with C3-fold symmetry’ Section, topological Weyl fermions are mainly attributed to the presence of Sn-centered Co SMR structural layers. Studies showed that the Fermi arc states serve as catalytic centers for the oxygen evolution process, making bonding and electron transfer more efficient through the partially filled orbitals. Although the surface area of a bulk single crystal of SMR Co_3_Sn_2_S_2_ is much smaller than that of Co-based nanostructured catalysts, its catalytic OER performance is impressive.

In addition to water splitting, another SMR material, PdTe_2_, was recently demonstrated to be a good electrocatalyst for the ethanol oxidation reaction (EOR) [124]. It is a topological Dirac semimetal and superconductor containing a SMR structural layer with C3-fold symmetry consisting of Pd or Te atoms, which was exfoliated into nanosheets. When the material was loaded onto carbon fiber paper for use as an EOR electrocatalyst, its performance was five times that of commercial Pd black.

**Figure 7.**
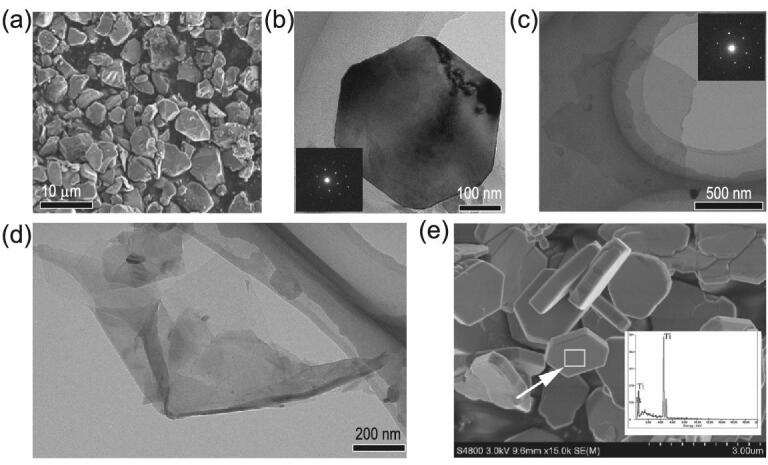
TiB_2_ crystals with different morphologies and sizes. Morphology and structure evolution of the crystalline TiB_2_ during the acidic hydrothermal process: (a) SEM image of pristine TiB_2_ (its particle size from 2 to 14 μm, Aldrich); (b–d) TEM images of three typical TiB_2_ hexagonal facets, thin platelets and ultrathin sheets derived from pristine TiB_2_ under the acidic hydrothermal process with a duration of around 4 h (reproduced with permission [125], Copyright 2009, the Royal Society of Chemistry). (e) SEM image and EDS pattern of TiB_2_ powders (reproduced with permission [126], Copyright 2019, American Scientific Publisher).

These examples strongly suggest the advantages of SMR materials with stable topologically protected non-trivial surface states that are exposed on the favorable facets to give outstanding HER and OER activities by providing more efficient electron transfer and/or binding interactions. However, their activities are greatly restricted by the large size and low surface area of the micro- and even larger crystals studied. Such large crystals cannot currently be avoided because of very high temperature (∼1300°C) needed for their preparation. To fully release the ability of SMR materials, developing efficient fabrication routes to produce them with selective exposure of a large favorable surface containing topologically protected surface states is therefore necessary. A top-down preparation method is advantageous for this purpose. Taking TiB_2_ (another AlB_2_-type material having Dirac nodal lines) as an example, the hydrothermal derivation of some well-defined hexagonal thin platelets and ultrathin sheets of TiB_2_ from commercial TiB_2_ crystals with a size of 2–14 μm (Fig. 7a–d) suggests a way of obtaining nanostructured TiB_2_ materials with a large surface area [125]. In addition, the large basal surfaces of these structures are (001) facets, where the topologically protected surface states have the largest exposure to reactants. Recent progress in the direct synthesis of well-defined TiB_2_ crystals with a hexagonal morphology has been reported (Fig. 7e) [126].

## REMARKS AND PERSPECTIVES

We have elucidated the relationships between the compositions and novel properties of several well-known material systems that contain six-membered rings and our analysis strongly points to a broad class of interesting materials, which we call SMR inorganic materials. These materials have always been important to research in the fields of materials science and condensed matter physics, and many of them have already become milestones in the history of materials development, showing great potential for next-generation, miniature, high-fault-tolerance and smart multi-functional devices in the fields of quantum, information, energy and the environment. First, most of these materials require the participation of *s*,*p* non-metallic or *s*,*p* metallic elements, which are highly abundant on Earth, and are both cheap and environmentally friendly. Second, SMR materials can have a wide range of compositions, from simple substances made of only one element, to binary and ternary compounds, and even multi-component alloys, and have a wide range of unique physical, chemical and mechanical properties. They can be wide-bandgap insulators, semiconductors, semimetals or metals. Third, most SMR materials can be easily prepared, for example by cleavage of bulk layered materials or even from earth-abundant layered mineral resources [127], suitable for a large variety of products.

Based on the above, we believe that they are an interesting class of materials that deserve more attention and further systematic investigation. This is just the beginning for SMR materials. Certainly, there is plenty of scope for using the SMR structural unit to design materials with unique properties, to discover new physics and to seek intriguing applications. However, the key bottleneck lies in the lack of fundamental knowledge of the basic principles underlying such materials and insufficient systematic research on their properties. We must establish a basic theory of the properties of these materials and understand any SMR correlations between these properties and the make-up of their basic SMR units, their symmetries, and their electron and phonon configurations to determine what materials are of interest and where to find them.

### Where to discover new SMR materials

We suggest the main focus should be on materials containing non-metal *s*,*p* and metal *s*,*p* electrons. In principle, SMR materials most frequently have an SMR structure consisting of *s*,*p* non-metallic elements, mainly because they easily form covalently bonded rings. From the thermodynamic point of view, the formation of covalent bonds requires very high energy. Metal and non-metal reactions are usually exothermic and provide enough energy for formation of covalent SMR structures of non-metallic *s*,*p* atoms alone or with some metallic elements. For instance, in many metallic borides, carbides, nitrides and sulfides, one can find SMR structural layers, and these materials should be the subject of further research.

**Figure 8.**
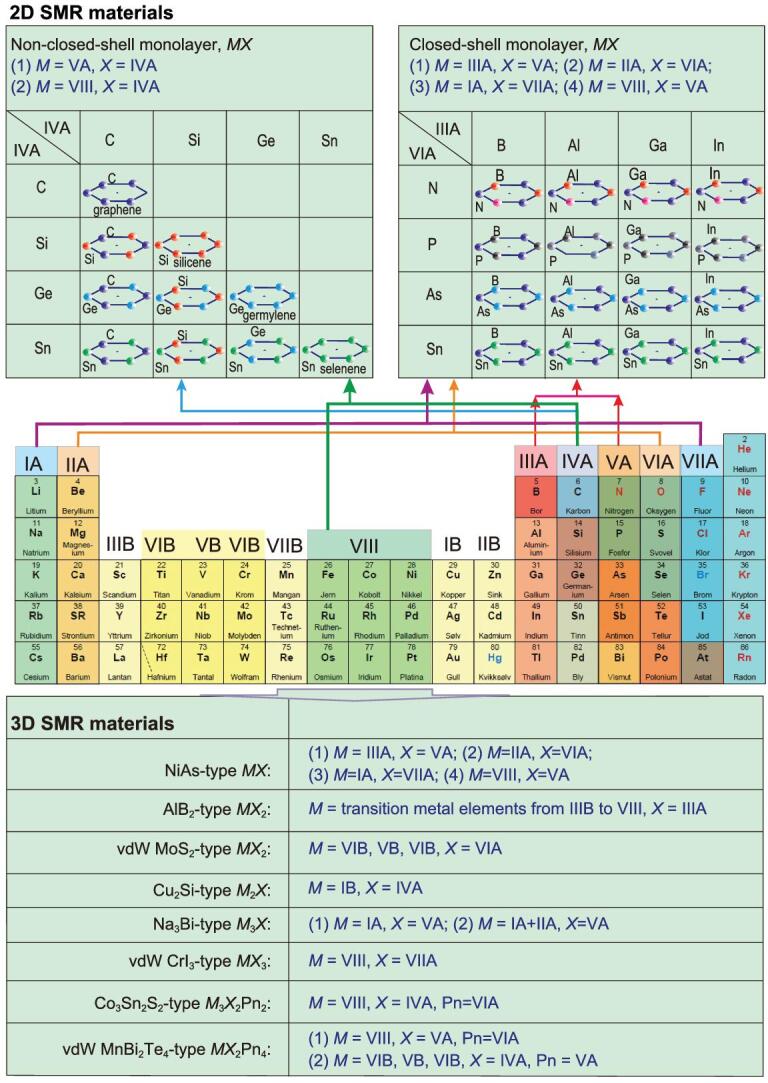
Plenty of room to exploit SMR materials from the atomic periodic table and some selected predictions. Upper panels: two representative examples of graphene and *h*-BN to illustrate various possible compositions. Left upper panel: 2D SMR materials in a non-closed-shell electronic configuration with a monolayer SMR structure among the IVA-group *s*,*p* elements. Right upper panel: 2D SMR materials in a closed-shell electronic configuration with a monolayer SMR structure between the VIA-group and the IIIA-group *s*,*p* elements. Middle panel: a specified atomic periodic table to guide readers as to how these compositions connect to SMR materials. Lower table: possible 3D SMR materials including van der Waals layered 3D materials with eight selected representative examples of NiAl-type, AlB_2_-type, vdW MoS_2_-type, Cu_2_Si-type, Na_3_Bi-type, vdW CrI_3_-type, Co_3_Sn_2_S_2_-type and vdW MnBi_2_Te_4_-type SMR materials.

Taking 2D monolayer SMR materials as examples, we expect to discover many new materials similar to graphene and *h*-BN (Fig. 8). Initially, attention needs to be paid to elements with a non-closed-shell electronic configuration, similar to that of graphene. IVA-group elements, with an outer *s*^2^*p*^2^ electronic configuration, have been used to synthesize materials with an SMR structure, such as silicene, germylene, selinene and plumbylene [128]. This is mainly because the outmost electronic configuration (s^2^p^2^) of the IVA-group elements are suitable for *sp*^2^ hybridization in an SMR structure. This kind of SMR structure can be extended from simple substances of the IVA-group elements into their binary compounds by combining two different IVA-group elements, e.g. 2D C-Si, C-Ge, C-Sn, C-Pb, Ge-Si, Ge-Sn, Ge-Pb and Sn-Pb, as shown in Fig. 8. According to the electronic configuration, it seems likely that these binary flat or buckled SMR 2D materials can be stabilized in *sp*^2^ or quasi-*sp*^2^ electronic hybridizations. Similar to monolayer *h*-BN, we can expect many similar combinations between VA-group and IIIA-group elements to form the closed-shell electron configuration of *MX* (*M* = IIIA-group B, Al, Ga, In; *X* = VA-group N, P, As, Sb, Bi) shown in Fig. 8. In fact, with the closed-shell electron configuration, *MX* can also be extended to other binary combinations of (i) IIA-group and VIA-group elements (*M* = Be, Mg, Ca, Sr, Ba; *X* = VIA-group O, Se, Se, Te, Po) and (ii) VIII transition metals and VA-group elements. In addition, all these monolayer SMR materials could be stacked to form 3D SMR materials. For instance, the SMR family of Be*X* compounds (*X* = VIA-group elements including O, S, Se, and Te) was experimentally characterized to have the space group *P*6_3_/mmc (No. 194) with a Be atom at the 2*a Wyckoff* site (0, 0, 0) and an *X* atom at the 2*c Wyckoff* site (1/3, 2/3, 1/4), which is isostructural to all other NiAs-type materials [129]. Following the same rule, more SMR *MX* compounds can be formed or predicted, with eight representative examples shown in Fig. 8. There are, of course, many other possibilities.

### How to discover new SMR materials

We suggest a strategy combining computational prediction and experimental verification to discover new SMR materials, according to the space groups and point groups from the angle of symmetry associated with the SMR structural unit. We suggest four steps that are needed to achieve this. First, the SMR structural unit provides a clear indication of how to innovatively combine possible elements in the periodic table. To begin one screens possible compositions according to elements and compositions in Fig. 8. As an example, if one wants to discover a ferromagnetic Weyl metal, it may be possible to find such a material among SMR M_3_X_2_*Pn*_2_ materials (*M* = 3d VIII: Fe, Co, Ni; X = IVA and *Pn* = VIA elements). Second, as illustrated in Fig. 4**,** the combinations among A1, A2 and A3 atoms provide crucial implications associated with symmetries to specify new SMR materials. With this framework, one needs to develop high-throughput computation coupled with large amounts of data and machine learning technologies to rapidly search for the possible existence of both 2D and 3D SMR materials. The computations should further screen the thermodynamic and dynamical stabilities of candidates and elucidate their physical, chemical and mechanical properties. Third, on the basis of these computations, one should compile a large amount of data on SMR materials, from their SMR structural units to their key properties, and then using machine learning and AI technologies effectively classify the materials into different types, to predict their properties, and to exploit potential applications. Fourth, with the aid of computation-driven data, the design of SMR materials may become possible and experiments will be required to verify theoretical predictions including the synthesis and characterization of SMR materials and the verification of their properties and potential uses.

### Which directions to be focused on SMR materials

We must emphasize that many aspects of SMR materials need to be further investigated, both theoretically and experimentally. Given that these are too numerous for all to be explored, we suggest the following three main aspects as the first steps.

#### SMR materials as platforms to discover new physics

SMR materials capture the essence of the inequivalent momentum, *K* and *K*′, the six corners of the first BZ. One of their intriguing physical properties is that the conduction band bottom and the valence band top exactly touch each other at these two points to form a Dirac cone in a linear correlation in graphene, and in monolayer MoS_2_ its electronic structure shows an energy-minimum electronic valley around them as detailed in ‘2D SMR materials’ Section. One even defines a valley quantum number as a property of an electron inside a crystal, which is correlated with the electronic momentum space. It is well known that the control of the spin degree of freedom allows manipulation of information and storage, which is a core of the field of spintronics. In similarity to spin manipulation, utilizing the valley degree of freedom, one may manipulate information in coupling with the valley states. In monolayer MoS_2_ the presence of SOC splits off the valence valleys at both *K* and *K*′ with opposite phase factors, which allows access to the valley degree of freedom using circularly polarized light [47] by a spin-valley coupling effect, as shown in Fig. 2c. In fact, once this kind of spin-valley coupling effect is combined with the abundant topological phenomena of SMR materials, rather than only with the previously known semiconductor MoS_2_, the fundamental physics and functionalities will be greater. For instance, if topological Dirac semimetals are combined with the spin-valley coupling interactions in SMR materials, one may expect the coexistence of bulk Dirac cones, topologically protected non-trivial surface (or edge) states and the spin-valley coupling interactions. This combination provides potential to control and manipulate the interplay between conducting surface (or edge) states and the spin-valley coupling using an electric field, a magnetic field and mechanical strain. This could be useful in low-power-consumption electronic, spintronic and valleytronic devices coupled with the edge currents of topological SMRs.

Another intriguing physical phenomenon lies in the survival of magnetic ordering interaction between atoms at the six corners of the SMR structures, as illustrated in the monolayer CrI_3_ and Cr_2_Ge_2_Te_6_ ferromagnetic semiconductors. These magnetic orderings add a new dimension to the physics of SMR materials, as revealed by the ferromagnetic Weyl metal Co_3_Sn_2_S_2_. Coupling between the topology and magnetic spin ordering have already shown a very large intrinsic anomalous Hall effect, as discussed in ‘3D SMR materials’ Section. Furthermore, it is possible to have the interactions between the magnetic spin ordering of atoms at the six corners of the SMR structure and the spin-valley coupling effects. Hence, these SMR materials may also provide the opportunity to construct various magnetic textures or hybrid devices with exotic quantum-mechanical properties, in particular, in the field of spin-based electronics and information technology.

#### SMR materials as high-performance catalysts for energy conversion

As discussed in ‘3D SMR materials for water splitting catalysis’ Section, SMR materials are candidates for various catalysis processes, not only for water splitting but also for other reactions, such as CO_2_ reduction and nitrogen fixation. This is mainly because such activity is closely related to the surface electronic structures of the catalysts, such as the surface (edge) states and surface (edge) atomic termination. From the viewpoint of traditional catalyst design, active sites are very important, and these can be provided by surface states derived from dangling bonds, vacancies and chemical dopants in bulk materials, as well as unsaturated electronic states from edges or surfaces of nanoparticles or 2D materials. Different from traditional design, SMR materials can potentially serve as catalysts because of the existence of topological electronic structures, including topological insulators, topological semimetals and topological nodal-line metals. Their bulk topological features induce stable metallic non-trivial surface states (e.g. helical surface (edge) states, Fermi arc states, and drumhead-like non-trivial states), which cover all possible surfaces of the material. In particular, SMR materials with potential as high-performance catalysts exhibit three main factors: (i) strongly active sites resulting from non-destroyable, highly localized and chemically active non-trivial surface states, which are resistant to surface modification, defects or other scattering; (ii) fast charge transfer originating from the high mobility of carriers associated with the presence of Dirac or Weyl cones and nodal lines and the lock-up electron spin significantly depressing backscattering and the Anderson localization of conduction electrons; (iii) a stable supply of carriers because the non-trivial surface states have to connect with bulk Dirac cones, Weyl cones or nodal-line states. Therefore, topological SMR materials such as Dirac semimetals, Weyl semimetals and topological nodal line semimetals can be good candidates for electrocatalysis or co-catalysts of photocatalysis. We believe that research on SMR materials will be a promising direction to design high-performance catalysts in combination with chemical reactions, as well documented most recently in a series of topological materials, theoretically or experimentally [115–117,124,130–139].

#### SMR materials as ultra-high temperature ceramics

The design of components for next-generation space vehicles, rocket nozzle inserts, and nose cones or leading edges for hypersonic aerospace vehicles requires novel materials, in particular, high melting-point ceramics with improved structural stability and ablation resistance. As discussed in ‘SMR-related chemical and mechanical properties’ Section, SMR materials with non-metallic *s*,*p* elements and refractory metallic elements have natural advantages to satisfy these requirements because they have strong covalent *sp*^2^ hybridization and an unusual combination of physiochemical properties, including high corrosion/oxidation resistance, chemical inertness, high thermal conductivity and extremely high melting points. Further theoretical progress on the design of novel SMR materials with superior mechanical properties, requires a deeper understanding of the formation of strong covalent bonding frameworks and of high cohesive energies. On the experimental side, perhaps the most important task is the engineering of high-melting-point SMR materials with non-metallic *s*,*p* elements. Besides ultra-high temperature ceramic composites (e.g. ZrB_2_-based ceramic composites), attractive possibilities are, on the one hand, the discovery of novel SMR materials (e.g. borides, nitrides and oxides) with much higher melting points and superior oxidation-resistance at ultra-high temperatures and, on the other hand, the innovative design of high-entropy transition metal borides, nitrides and oxides with the SMR structural unit.

There are certainly more aspects of SMR materials which are not mentioned in this discussion. These include covalent SMR materials for effective corrosion protection (e.g. graphene and *h*-BN) and the possibilities of using ionic SMR materials in batteries and for ion transport in energy storage and chemical separation. Another interesting topic is extension of the SMR structural unit. First, the SMR structural unit can be extended into a general structural unit by varying six edges or atoms at its six zeniths of the corners of SMRs. For instance, the six identical two-carbon-atom bonding edges of the ideal SMR structural unit in graphene can be replaced by six long four-carbon-atom bonding edges in α-graphyne [140] or by alternating two different kinds of edges of three long four-carbon-atom edges and three short two-carbon-atom edges in β-graphyne [140]. Of course, the atoms at the six zeniths of SMR can be replaced by small atomic clusters. Second, SMR can be closely related to the presence of five/seven (four/eight, three/nine, eleven/three)-membered-ring structures created by rotating bonds and typical patterning defects in SMR structures (i.e. the manipulation of Thrower-Stone-Wales defects [141] and the inverse Thrower-Stone-Wales defects to create various other 2D carbon allotropes such as *T*-Graphene [142], on the basis of graphene [143]). Third, a 2D SMR material can be rolled into a 1D tube material. For instance, a 1D carbon nanotube is constructed by rolling up a 2D SMR graphene. Although massless Dirac cone occurs in 2D SMR materials, the electronic structure of 1D nanotube [144–147] indeed depends sensitively on the wrapping angles and diameters of SMR structures.

**Figure 9.**
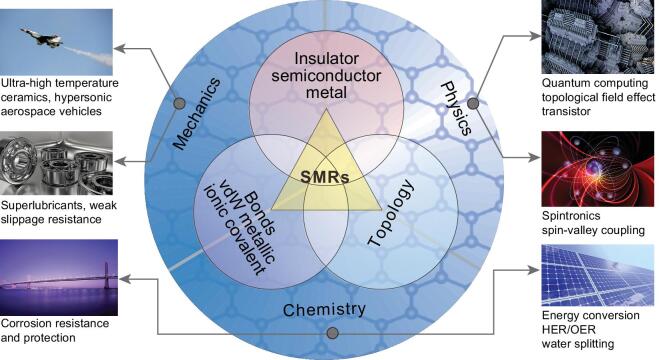
The future of SMR materials and their interplay among chemical bonds, electron and phonon, and topology in coupling with the SMR structural gene across wide types of compositions and structures. Several SMR-driven functionalities and applications are listed: (1) quantum computing, topological field effect transistor, spintronics and valleytronics in the field of physics; (2) energy conversion for various chemical processes and corrosion-resistant behaviors and their protection in the field of chemistry; (3) ultra-high temperature ceramics, supersonic aerospace vehicles in extreme conditions and environments, as well as super-lubricants in mechanics.

Finally, we must point out that this new concept of SMR materials emphasizes a basic structural unit that gives rise to an abundant family of materials, which deserve widespread, systematic investigation. As illustrated in Fig. 9, on the basis of the SMR structural unit, the interplay among various bonding, electron and phonon structures may find use in many different applications involving their electronic properties (quantum computing, spintronics and valleytronics), chemical properties (catalysis and corrosion protection) and mechanical properties (super-high temperature ceramics and long-term lubricants). In addition, heterostructured SMR materials constructed from the many individual SMR materials will definitely further widen these potential applications and many others in the fields of quantum science, information and energy technology, environmental science, as well as space exploration.

## Supplementary Material

nwaa248_Supplemental_Files
