# Supplementary material for: Six-membered-ring inorganic materials: definition and prospects
Source: Natl Sci Rev. 2020 Sep 28;8(1):nwaa248. doi: 10.1093/nsr/nwaa248 (PMC8294346; doi:10.1093/nsr/nwaa248)
Supplement: nwaa248_Supplemental_Files [file nwaa248_supplemental_files.docx]

**Teaser text**

The authors defined SMR materials, which possess extensive six-membered-ring structural units and rich structural lattices, chemistry and physics, suitable for niche applications in next-generation information technology, renewable energy, space, etc.
